# Deep Learning-Based Prognostics and Health Management Model for Pilot-Operated Cryogenic Safety Valves

**DOI:** 10.3390/s24061814

**Published:** 2024-03-12

**Authors:** Minho Kim, Hansaem Seong, Dohyun Kim

**Affiliations:** 1 Department of Computer and Information Engineering, Catholic University of Pusan, Busan 46252, Republic of Korea; minho@cup.ac.kr; 2 DH Controls Co., Ltd., Busan 46747, Republic of Korea; seong.hansaem@dohn.kr

**Keywords:** prognostics and health management, PHM model, data-driven prediction models, anomaly detection, real-time monitoring, safety valves

## Abstract

This paper highlights the significance of safety and reliability in modern industries, particularly in sectors like petroleum and LNG, where safety valves play a critical role in ensuring system safety under extreme conditions. To enhance the reliability of these valves, this study aims to develop a deep learning-based prognostics and health management (PHM) model. Past empirical methods have limitations, driving the need for data-driven prediction models. The proposed model monitors safety valve performance, detects anomalies in real time, and prevents accidents caused by system failures. The research focuses on collecting sensor data, analyzing trends for lifespan prediction and normal operation, and integrating data for anomaly detection. This study compares related research and existing models, presents detailed results, and discusses future research directions. Ultimately, this research contributes to the safe operation and anomaly detection of pilot-operated cryogenic safety valves in industrial settings.

## 1. Introduction

In industries such as petroleum and liquefied natural gas (LNG), safety and reliability are not just operational goals but are critical for preventing catastrophic failures. Safety valves serve as essential components by acting as a fail-safe to control pressure and prevent accidents, thereby contributing significantly to the overall system safety. These valves are particularly crucial in managing operational risks in extreme environments and low-temperature conditions, where material and mechanical failures could have severe consequences. Hence, the development of reliability prediction and anomaly detection technologies is essential. Historically, empirical methods relied heavily on historical data and heuristic analysis, presenting limitations in accuracy, adaptability, and real-time analysis. These methods often failed to capture the complex behaviors of safety valves in dynamic operational environments. Consequently, data-driven prediction models have gained significant attention recently, particularly with the advancements in machine learning and deep learning. This study presents a novel approach to generating failure data for PHM systems. Furthermore, it provides a comprehensive comparison of time series data analysis algorithms, identifying the most suitable model for enhancing the reliability of safety valves.

**Figure 1:**
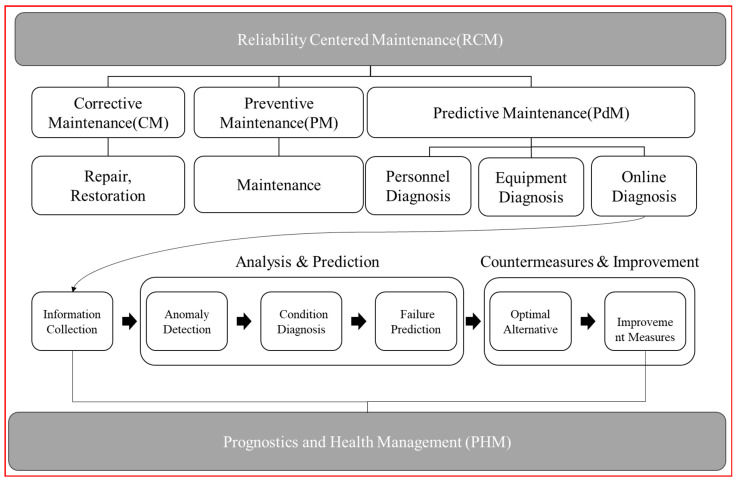
Relationship between reliability-centered maintenance (RCM) and prognostics and health management (PHM).

The prognostics and health management (PHM) of safety valves is recognized as a crucial factor contributing to safe operation and enhanced productivity in industrial facilities [1,2]. While regular maintenance and preventive inspections are vital for maintaining system safety [3], the developed PHM model utilizes real-time monitoring and machine learning algorithms to predict failures and anomalies in safety valves, enhancing reliability through proactive maintenance and timely interventions [4]. Such a model monitors the performance and condition of operating systems, facilitating early anomaly detection to prevent potential risks and enabling timely actions [5,6]. Safety valves may be exposed to unpredictable state changes due to behavioral pattern variations or irregular environmental changes. In such situations, it is essential to predict integrity using a reliable predictive model and conduct proactive inspections and maintenance to prevent accidents caused by system failures. Figure 1 shows the relationship between RCM and PHM.

This paper introduces a novel approach to enhancing the reliability and safety of pilot-operated cryogenic safety valves through the strategic collection and analysis of sensor data. Central to our research is the hypothesis that synthetically generated failure data, based on meticulously designed scenarios, can accurately replicate real-world failure conditions. Furthermore, it is posited that among the various time series data analysis algorithms available, a specific algorithm stands out for its suitability in precisely predicting safety valve failures. Guided by this hypothesis, this study not only focuses on developing advanced functionalities for lifespan prediction and normal operation discrimination but also on the real-time integration of acquired data for robust anomaly detection. The proposed deep learning-based prognostics and health management (PHM) model leverages diverse features extracted from raw sensor data, enabling the distinction between normal and abnormal operational states and the prediction of potential failures within defined timeframes. This approach reflects a significant methodological innovation, aiming to overcome the limitations of traditional empirical methods by providing a more accurate, adaptable, and predictive framework for ensuring the safety and reliability of these critical components.

This paper will be structured as follows: Firstly, in Section 2, trends in relevant research and existing safety valve prediction models will be investigated and compared. Section 3 will describe data collection and preprocessing, while Section 4 will present a detailed explanation and results of the developed deep learning-based PHM model. Finally, Section 5 will discuss the results, conclusions, and future research directions.

This research proposes a deep learning-based PHM model for pilot-operated cryogenic safety valves, contributing to safe operation and anomaly detection in industrial settings.

## 2. Related Research

### 2.1. Traditional Approaches for Robustness Prediction and Management

**Figure 2:**
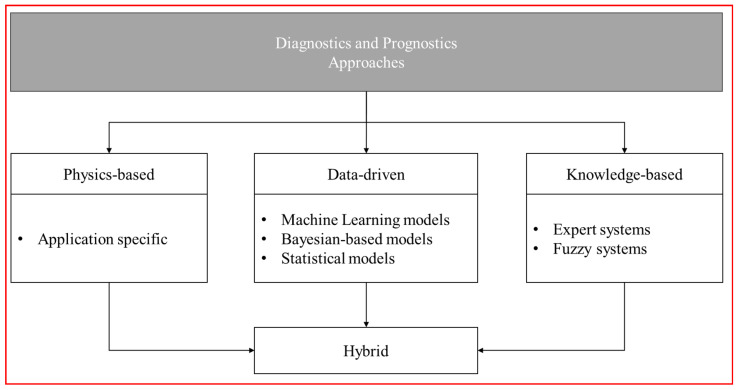
Approach for prognostics and health management (PHM).

In the past, reliability-centered maintenance (RCM) heavily relied on corrective maintenance (CM) after damage and failure occurred. However, this approach was not applicable to highly reliable systems as mentioned earlier, and now, it relies on preventive maintenance (PM) through regular maintenance. Nevertheless, preventive maintenance involves performing maintenance regardless of the actual fault level, resulting in frequent downtime and high costs for component replacement. To address these issues, prognostics and health management (PHM) technology emerged. PHM technology aims to achieve predictive maintenance (PdM), which involves performing maintenance only when necessary, thus minimizing failures and significantly reducing maintenance costs. Figure 2 shows an approach for prognostics and PHM.

PHM technology enables predictive maintenance through stages of information collection, anomaly detection, condition diagnosis, and failure prediction. In the information collection stage, data are collected from sensors such as temperature, humidity, and acceleration and characteristic signals related to faults are extracted through noise removal and signal processing. In the anomaly detection stage, the collected characteristic signals are measured against normal operation data to identify deviations, triggering anomaly alerts when they fall outside the normal range. In the condition diagnosis stage, the state information is used to comprehensively evaluate the health status of the process/asset, including the presence of anomalies or faults and their severity, providing necessary information for normalization. Finally, in the failure prediction stage, the remaining useful life (RUL) is predicted based on the health assessment results to plan maintenance schedules. Fault prediction is a crucial element of PHM, which can be broadly categorized into a physics-based approach, data-driven approach, and knowledge-based approach, and a hybrid approach that combines them is also used:

- Physics-based approach: This approach is based on understanding the physical aspects of failure (e.g., fault growth or degradation) and is applicable when an accurate mathematical model for the target can be used [7]. It evaluates the residuals (errors) between actual performance measurements and predictions from the mathematical model to determine the presence of faults. Statistical techniques are used to evaluate residuals, assuming that significant residuals indicate malfunctions, while small residuals may result from noise or modeling errors [8]. Bayesian hypothesis testing and probabilistic RUL predictions are used to assess the superiority of the degradation model and evaluate errors/biases in measured data [9].
- Data-driven approach: In contrast to the physics-based approach, data-driven approaches do not always rely on reliable physical models, making them suitable when such models are unavailable. These approaches focus on learning mathematical models based on correlations and dependencies between the output variables of interest and available variables [10,11]. Data quantity and quality are crucial, and if sufficient data are available, this approach can be implemented without requiring a physical model [12].
- Knowledge-based approach: Knowledge-based models rely on comparing predefined failure libraries and observed situations to provide intuitive results based on engineering experience and historical events [13]. Constructing such models is a challenging task and mostly relies on the expertise of domain experts [14]. However, recent research has been exploring the integration of knowledge-based models with other approaches through hybrid approaches to add flexibility to the modeling process, especially in complex systems.

Hybrid approaches are used to increase the reliability of fault prediction by combining the advantages of physics-based, data-driven, and knowledge-based approaches. They can be categorized into serial and parallel models, with each model performing parameter estimation for the physics model using a data-driven approach and learning residuals of the physics model through a data-driven model. Knowledge-based approaches involve knowledge bases expressed in rules, frames, predicate logic, etc., and inference engines based on domain experts’ expertise, including expert systems and fuzzy logic. Hybrid approaches add some flexibility to the modeling process, making them useful for complex systems [15]. In this paper, the data-driven approach is chosen to develop a robustness prediction management model for the cryogenic safety valve for the following reasons:

- Complexity of the system and physical model: Some complex systems may be challenging to construct accurate physical models for. Real system behavior is influenced by various physical factors and interactions, making it difficult to represent them accurately with mathematical models. Moreover, building a physical model often requires deep domain knowledge and extensive experimental data, which might be limited.
- Diversity and volume of data: Data-driven approaches can utilize diverse data collected from various sensors to model system behavior, reflecting interactions and influences that might be challenging to consider with a physics-based approach. Additionally, a large amount of data enables more sophisticated modeling and predictions. In the era of big data, the increase in data quantity and diversity makes data-driven approaches more important, as they can effectively analyze data to extract valuable information.
- Real-time prediction and adaptability: Some systems change rapidly and operate under different environments. In such cases, data-driven approaches are advantageous, as they can collect and analyze data in real time to update prediction models. Physical models may require repeated experiments and model reconstructions to apply changes due to system variations or external influences. However, data-driven approaches can continuously update and adapt models with new data, making them more suitable for real-time prediction and adaptability.

### 2.2. Trends in Data-Driven Fault Prediction Research

Over the past few decades, data-driven fault prediction research has focused on traditional machine learning methods such as support vector machines (SVMs), which rely on manually extracted features. However, these traditional methods had limitations due to the significant time and incomplete information required for feature extraction, hindering performance improvement. In recent years, artificial neural networks based on high-dimensional vector representation have shown outstanding performance in various fields, including fault prediction. Deep learning models such as convolutional neural networks (CNNs), recurrent neural networks (RNNs), recursive neural networks (recursive NNs), etc., are being used in data-driven approaches.

The accuracy of data-driven approaches depends on the quality and quantity of the data used. However, determining how much data is necessary without detailed knowledge of the target system can be challenging. Data-driven approaches involve implementing mathematical models based on various algorithms and using mathematical functions and available information for modeling. Additionally, simulations are used to generate artificial data for model training and evaluation. These data-driven approaches are quick and easy to implement, utilizing data mining or machine learning packages for straightforward use. Moreover, with sufficient data, new relationships can be discovered and models can be constructed, considering all relationships without bias. However, data-driven methods require a considerable amount of data containing as many fault states as possible for the same or similar systems, and interpretation may be difficult, as prediction results are not directly related to physical knowledge, posing potential risks.

In data-driven fault prediction, sensor data collected from equipment or products are used to evaluate the current state, detect anomalies, and predict the remaining useful life. Sensor data consist of time series data containing continuous observations of values such as temperature, pressure, voltage, vibration, etc., with meaningful temporal patterns. Data-driven fault prediction utilizes these time series data to discover trends, seasonality, periodicity, etc., and models them to predict future values. For instance, fault prediction algorithms for gearboxes use varying peak vibration frequencies and sizes fitted to the time series data to predict future values, comparing them with threshold values that define the normal operation to predict the occurrence and timing of faults. Recently, deep learning models for time series data analysis are broadly categorized into RNN-based models (e.g., LSTM or GRU), Transformer-based models that use self-attention mechanisms to replace RNN-based models, and TCN-based models, which are CNN-applied to time series data to better reflect local correlations.

- RNN-based models: These models store past information in memory and update it with each new input, but models like LSTM have issues with slow calculation speed and difficulty in representing long-term dependencies between distant data points.
- Transformer-based models: Transformer-based models use self-attention mechanisms to replace RNN-based models, and models like the Transformer-Encoder have proposed efficient and effective methodologies to improve prediction performance. However, some Transformer-based models have significant drawbacks in size and computational complexity, leading to ongoing research to address these limitations [16,17,18].
- TCN-based models: TCN designs CNN principles applied to time series data to better reflect local correlations. TCN is more parallelizable than RNN-based models and can avoid the vanishing gradient problem. However, it consumes more memory [19,20].

In this paper, a data-driven approach is analyzed to select the optimal model for robustness prediction management of cryogenic safety valves. The comparison encompasses deep learning models including convolutional neural networks (CNNs), recurrent neural networks (RNNs), and Transformer-based models. Deep learning models are chosen for their advanced capability to handle complex, nonlinear patterns and high-dimensional data typical in sensor data analysis. Among deep learning approaches, RNN-based models, Transformer-based models, and temporal convolutional network (TCN)-based models are evaluated for their suitability in processing time series data. This assessment aims to determine which model best addresses the intricate relationships within time series data, automatically extracting relevant features without the need for manual feature engineering. The inherent ability of these models to learn from vast amounts of operational data positions deep learning as the preferred methodology for enhancing the predictive accuracy and reliability of the prognostics and health management (PHM) system. The comparison not only highlights the shortcomings of traditional machine learning methods, which often rely on manual feature selection, but also underscores the efficiency and effectiveness of deep learning models in real-time anomaly detection and system behavior analysis.

## 3. Data Collection and Preprocessing through Testbed Construction

In this chapter, we will explain the process of constructing a testbed for the cryogenic safety valve and the data collection and preprocessing procedures, including measuring various variables such as pressure, flow rate, and noise using sensors.

### 3.1. Testbed Construction for Data Collection

**Figure 3:**
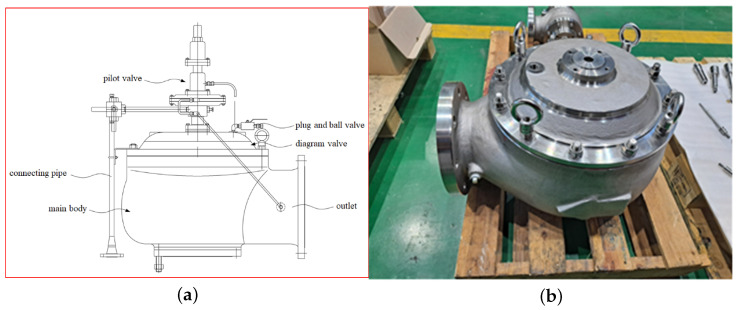
Drawings and prototypes of pilot-operated cryogenic safety valves. (**a**) Drawing, (**b**) prototype.

To evaluate the performance and reliability of the cryogenic safety valve, we constructed a testbed. The testbed simulates conditions similar to the actual operating environment, enabling us to collect and analyze data related to the operation of the safety valve. The safety valve used in this testbed is a pilot-operated relief valve, which is essential safety equipment used in facilities such as LNG-powered ships, LNG transport vessels, and LNG bunkering equipment. The pilot-operated relief valve maintains a tightly closed state below the operating pressure using the pressure of the internal fluid but quickly opens when the operating pressure is reached. Once the valve is open, it rapidly closes after discharging the excess pressure, preventing unnecessary waste of internal fluid (vaporized natural gas). The safety valve produced for this study has a specification of 2 × 3 inches and consists of a main body that connects to the gas storage tank and the main body’s inlet, a diaphragm valve that opens and closes the inlet, and a pilot valve that operates the diaphragm valve. A seating is installed at the inlet to enhance the sealing performance, and the characteristic of this safety valve is the fine adjustment of the closing pressure through closing pressure regulation. Figure 3 shows drawings and prototypes of pilot-operated cryogenic safety valves.

**Figure 4:**
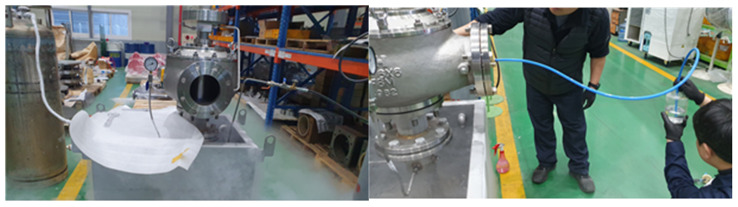
A view of the testbed for data collection.

The testbed utilizes various sensors to collect data related to the safety valve in real time. First, a pressure sensor is used to measure the internal pressure of the safety valve. This sensor detects and records pressure changes during the operation of the safety valve, providing information about its pressure state. Additionally, a flow rate sensor is used to measure the fluid flow passing through the safety valve. This sensor tracks the flow rate of the fluid and collects flow rate data of the safety valve. Figure 4 shows a view of the test bed for data collection.

However, the noise measurement sensor that occurs during the operation of the safety valve was excluded from the testbed. This is because the noise data could be obscured by other noises generated in the surrounding environment, making accurate measurements difficult. Noise generated during the operation of the safety valve is particularly sensitive in the cryogenic environment, and interference from ambient noise made it challenging to collect precise noise data. Therefore, we plan to evaluate the changes in the safety valve’s state and utilize the data obtained from other sensors for model training without using the noise measurement sensor. The data collected from the test bed provide rich information about the operation of the safety valve under normal and abnormal conditions. Based on these data, a predictive model will be developed to detect abnormal states of the safety valve and contribute to the safe and reliable operation of the system. In the future, there is anticipation for the construction of a smart monitoring system capable of monitoring the state of the safety valve and utilizing it for failure prediction in actual operating environments.

### 3.2. Data Collection for Normal and Abnormal States

**Figure 5:**
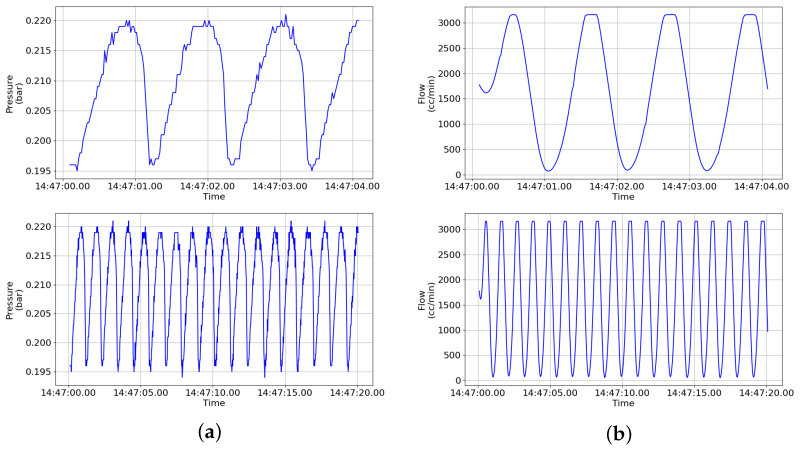
Examples of data collected from pressure and flow sensors. (**a**) Pressure, (**b**) flow.

Collecting data for abnormal states in real environments is often challenging. Abnormal states can occur unexpectedly, and their causes and patterns are diverse, making it difficult for them to occur with sufficient frequency in actual operating environments. Additionally, ensuring safety is a priority when abnormal states occur, which makes intentionally inducing abnormal states or damaging equipment inappropriate and risky. Figure 5 shows examples of data collected from pressure and flow sensors.

Due to these difficulties, researchers have had to artificially create abnormal states for data collection. To do this, simulation techniques are used to construct various abnormal state scenarios and generate data for those states. By artificially generating data through simulations, researchers can manipulate data for specific situations, provide accurate labeling, and use them for model training. In the case of safety valves, abnormal states were also simulated to generate data. Various situations, such as pressure release reduction due to foreign material buildup in the pilot line or insufficient differential pressure formation in the main valve, were simulated within the system. Data from these situations were measured and recorded to build the database. These artificially generated data were used for both training and evaluating the smart monitoring system, alongside normal state data.

**Figure 6:**
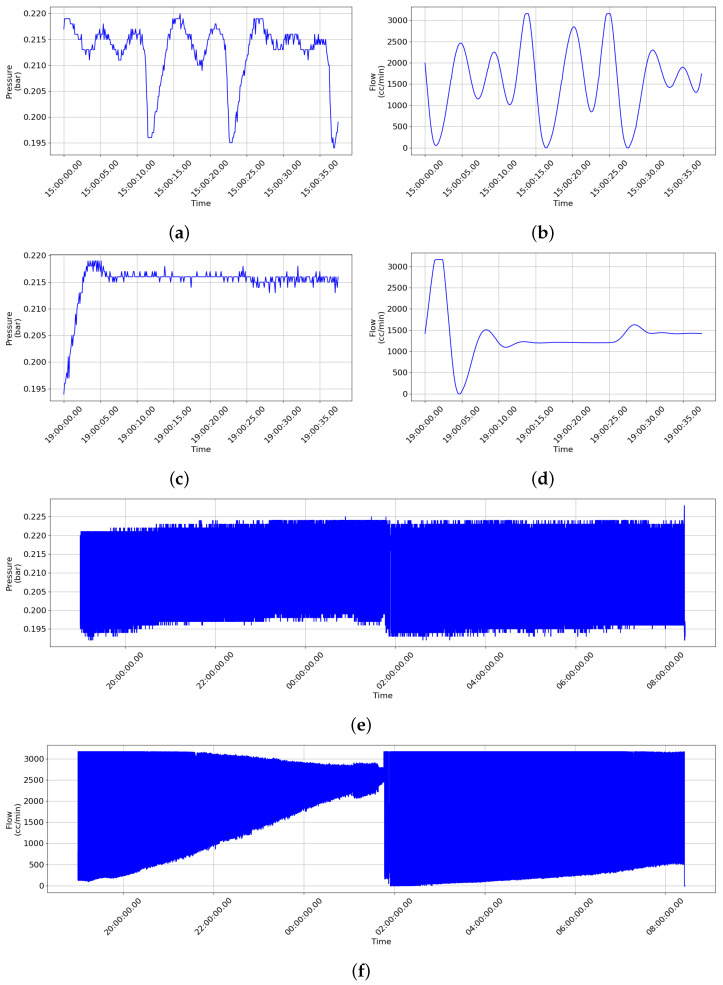
Examples of anomaly data collected in individual situations. (**a**) Abnormal case 1: pressure, (**b**) abnormal case 1: flow, (**c**) abnormal case 2: pressure, (**d**) abnormal case 2: flow, (**e**) abnormal case 3: pressure, (**f**) abnormal case 3: flow.

By using artificially generated abnormal state data, important information for evaluating the system’s stability and performance was obtained. Furthermore, developing and validating fault prediction models using these data greatly contributed to the development of a smart monitoring system that can operate safely and efficiently in real operating environments. Therefore, the implementation and analysis of data-driven approaches through artificially generated abnormal state data brought us closer to the development of a safe and reliable system. Figure 6 shows examples of anomaly data collected in individual situations.

During the data collection process for normal states, the safety valve was operated (opened) to discharge internal pressure up to 3100 L/min at approximately 0.195 bar. Additionally, at around 0.22 bar, the operation of the safety valve was stopped (closed) to prevent unnecessary pressure discharge. These normal state data reflect the typical operating conditions of the safety valve and are used for training and evaluating the smart monitoring system. During the data collection process for abnormal states, various situations are simulated to collect data reflecting abnormal operations of the safety valve. In Case 1, due to foreign material buildup in the pilot line, the pressure discharge to the pilot line is reduced and sufficient differential pressure is not formed in the upper and lower parts of the main valve, resulting in inadequate opening of the main valve. As a result, the intermittent phenomenon occurs where only half of the normal operating flow rate is discharged. In Case 2, the abnormal operation observed in Case 1 continues to occur. The pressure discharge remains reduced in the pilot line due to foreign material buildup, causing the main valve to remain in an unopenable state. In Case 3, due to foreign material buildup in the pilot line, the pressure discharge is reduced and the main valve remains unopenable until an abnormal operation eventually occurs. This abnormal operation state indicates that the valve, which normally exhibits 100% opening, fluctuates as follows: 0% → 99% → 5% … 90% → 80% → 90% → 80% during its operation.

## 4. Experiment and Evaluation

The PHM model proposed in this paper utilizes pressure and flow data generated during normal operation to predict abnormal behavior. Firstly, a model is trained to recognize normal operation patterns using pressure and flow data from normal operating conditions. The trained model is then used to monitor pressure and flow data in real time and generate predictions based on this input. If the error between the predictions generated by the model and the actual data exceeds a predefined threshold, it is identified as an abnormal state.

### 4.1. Data Preprocessing

**Figure 7:**
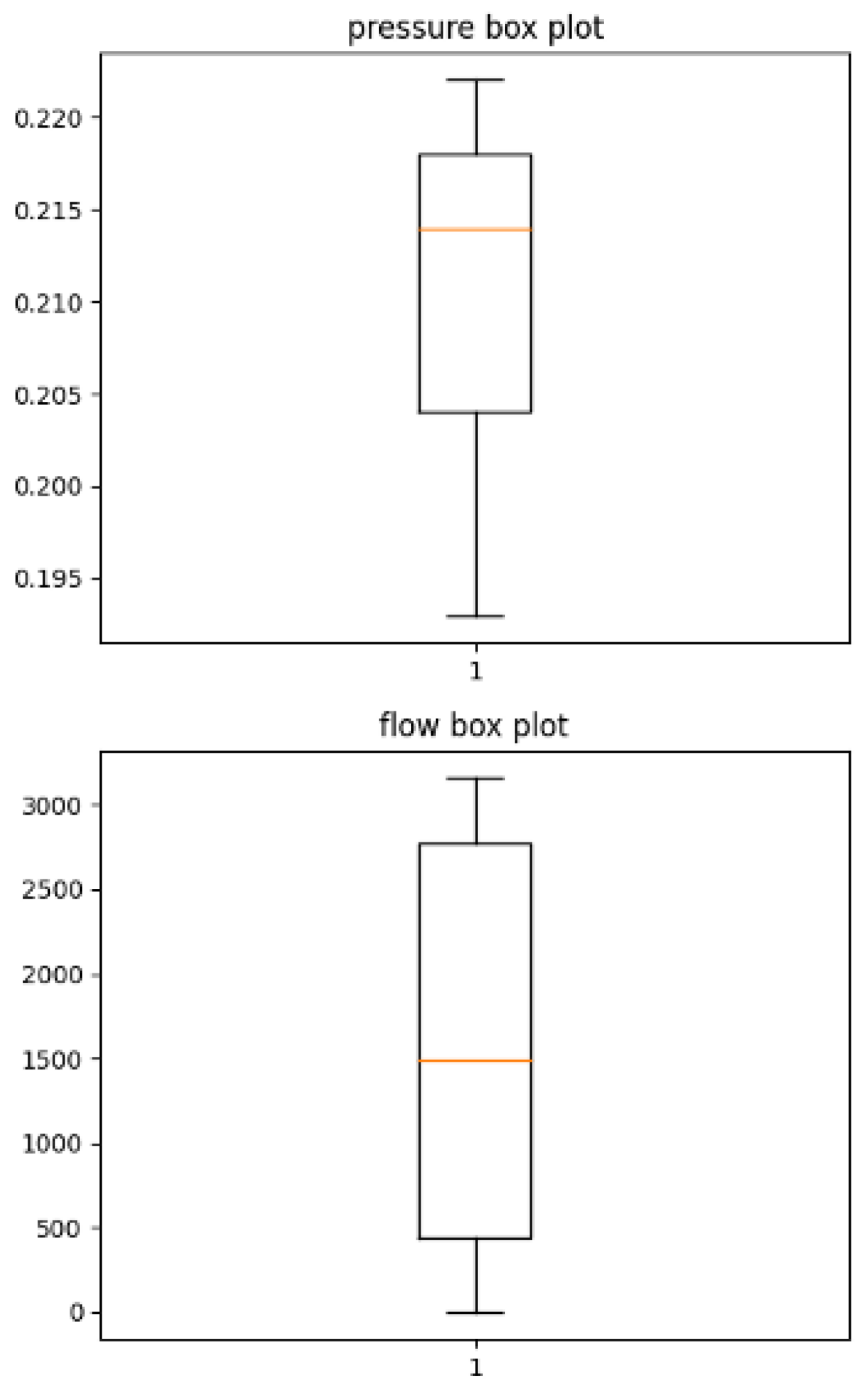
Box plots for pressure and flow to identify outliers.

When training time series analysis models on time series data, data normalization is a commonly recommended procedure in machine learning. In particular, since pressure and flow have different units and ranges, scaling each feature to the same range can enhance the model’s training convergence speed and generalization ability. There are primarily two methods commonly used for data normalization: min-max scaling and standardization. Min-max scaling is advantageous because it is easy to interpret, and it is not sensitive to outliers, as it scales the data based on the entire data range regardless of the presence of outliers. However, if the minimum and maximum values are heavily influenced by outliers, it can impact the scaling results significantly. On the other hand, standardization uses the mean and standard deviation to transform the data. This method can handle data distributions stably even when outliers are present, but it has the drawback of making the interpretation of scaled data more challenging. Figure 7 displays pressure and flow using a box plot.

A box plot consists of a box, representing the median (or the second quartile) of the data, and whiskers that depict the distribution of the data. The lower boundary of the box represents the first quartile (Q1), while the upper boundary represents the third quartile (Q3). The whiskers represent the range of the data and are typically drawn up to a distance of 1.5 times the interquartile range (IQR) from the box boundaries. Points outside the whiskers are usually considered outliers. As evident from Figure 7, there are no outliers in the pressure and flow data. Therefore, data preprocessing will proceed using the easily interpretable min-max normalization according to the following formula:

(1) $$x_{\mathrm{scaled}}=\frac{x-x_{\min}}{x_{\max}-x_{\min}}$$

This min-max normalization will make the data fall within a range of [0, 1], making them suitable for further analysis while preserving the interpretability of the data.

### 4.2. Experimental Environment and Results

Time series data are sequences of data points ordered chronologically and arranged at regular or irregular intervals. The prediction process involves modeling the series either based solely on past behavior (autoregressive) or by incorporating other external variables to forecast future values of the time series. To apply machine learning models to forecasting problems, a time series needs to be transformed into a matrix where each value is associated with a specific time window (known as lag). In the context of time series, the lag for a time step t is defined in terms of previous time steps’ series values. For example, lag 1 represents the value at time step t1, and lag m represents the value at time step tm. In univariate time series forecasting, a single time series is modeled as a linear or nonlinear combination of lags, where past values of the series are used to predict the future. In multivariate time series forecasting, a single model is used to simultaneously model two or more time series together. There are two distinct strategies for multivariate time series forecasting:

- Independent multi-series forecasting: in this approach, a single model is trained for all time series, but each time series remains independent of the others, meaning that past values of one series are not used as predictors of other series.
- Dependent multi-series forecasting (multivariate time series): here, all series are modeled together in a single model, considering that each time series depends not only on its past values but also on the past values of the other series.

**Figure 8:**
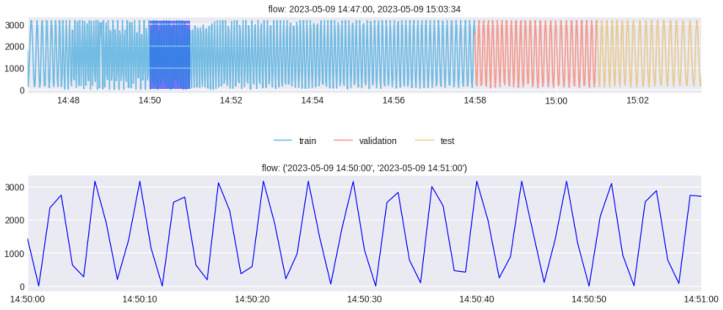
Train data split into training, validation, and evaluation datasets.

As described in Section 3, during the data construction process, pressure and flow are closely related. Therefore, it is reasonable to predict pressure through multivariate time series forecasting that utilizes both pressure and flow. Meanwhile, Figure 8 illustrates the division of pressure and flow data measured at intervals of 200 ms from 14:47 to 15:03 on 9 May 2023 into training, validation, and evaluation datasets.

The original data with a 200 ms interval contained more detailed information, but they were downsampled to a 1 s interval to reduce the data dimensionality for more efficient computations during model training and prediction. Through this downsampling, the train data were divided into a training set of 661 s, a validation set of 181 s, and an evaluation set of 155 s.

In this paper, as previously explained, data-driven approaches are compared and analyzed to select the optimal model for prognostic management of the safety valve’s integrity. Traditional machine learning methods from the linear regression family are compared and evaluated, as well as deep learning models like convolutional neural networks (CNNs), recurrent neural networks (RNNs), and Transformer-based models. For traditional linear regression methods, XGBoost was used, while for CNN models, SCINet, one of the temporal convolutional network (TCN) models designed for time series analysis, was employed. For RNN models, LSTM was utilized, and finally, for Transformer-based models, Informer was chosen.

Table 1 presents the prediction results for the flow rate. The experimental setup is identical to [20], with an input length of 72 s used to measure short-term prediction performance, forecasting 36 s into the future. As evident in Table 1, SCINet exhibited the best performance. This is attributed to SCINet’s robust performance on noisy real-world data, achieved through downsampling and interactive learning.

As demonstrated in Table 1’s results, SCINet, a model from the TCN family, showed the highest accuracy in predicting pressure changes under normal operational conditions. This outcome suggests that models well trained on normal operation data can effectively detect abnormal states; a discrepancy between the model’s predictions and actual observations signifies a deviation from normal behavior. The forthcoming experiment will detail an experiment designed to verify this capability, focusing on the model’s application in real-time monitoring to identify anomalies, thereby affirming its practical value in predictive maintenance and safety management.

Table 2 summarizes how accurately models trained on a dataset consisting of only normal operational data classify each on a dataset with a mixture of normal and abnormal states. The basic indicator for anomaly detection is when the deviation between the model’s predictions and the actual value is greater than a predefined threshold (pressure 0.1 bar).

Following the detailed analysis and results presented in Table 2, the experiment substantiates the hypothesis that models trained solely on normal operational data can discern between normal and abnormal operational states with a high degree of accuracy. This discernment is primarily facilitated by identifying instances where the predicted pressure deviates from the actual measurements beyond a predefined threshold, herein set at 0.1 bar. Such a threshold-based approach for anomaly detection highlights the model’s capacity to pinpoint discrepancies indicative of potential malfunctions or deviations from the expected operational parameters.

The precision, recall, and F1-scores for both normal and abnormal classifications, as outlined in Table 2, further elucidate the model’s efficiency and reliability in anomaly detection within mixed operational data. Notably, the high precision and recall rates for abnormal states underscore the model’s adeptness at identifying genuine anomalies, thereby minimizing the risks of overlooking critical operational irregularities that could lead to unforeseen downtime or safety hazards.

Moreover, the macro and micro averages reinforce the model’s overall performance robustness, indicating a balanced detection capability across both normal and abnormal states. Such balanced performance is imperative for predictive maintenance and safety management applications, where the cost of false negatives (unidentified anomalies) could significantly outweigh that of false positives (normal operations mistakenly flagged as anomalies).

The implications of these findings extend beyond the immediate scope of operational monitoring, offering potential pathways for enhancing predictive maintenance strategies. By leveraging models adept at distinguishing between normal and abnormal states based solely on normal operational data, organizations can implement more proactive and less intrusive maintenance routines. This proactive approach not only ensures operational efficiency but also contributes to the overarching goal of safety management by preemptively addressing issues before they escalate into critical failures.

In conclusion, the experiment validates the practical applicability of utilizing models trained on normal operational data for real-time anomaly detection. This validation not only affirms the theoretical underpinnings discussed earlier in the paper but also showcases the tangible benefits of integrating such models into the operational framework of predictive maintenance and safety management systems. Future work may explore the optimization of deviation thresholds and the incorporation of additional operational parameters to further enhance the model’s diagnostic capabilities, thereby broadening the horizon for automated anomaly detection in various industrial applications.

## 5. Discussion

This study addressed the challenge of obtaining failure data in data-driven prognostics and health management (PHM) by leveraging simulated data that replicate failure scenarios. Through comparing various models extensively utilized for time series data analysis, the most appropriate model for this domain was identified. It was experimentally demonstrated that predictive models can be trained using solely normal operation data, enabling the classification of operational statuses. The precision in anomaly detection could potentially be enhanced by fine-tuning the threshold settings between actual and predicted values based on expert experience.

However, the utilization of simulated data instead of real failure instances and the reliance on basic data preprocessing methods (i.e., removing missing values and outliers) are acknowledged as limitations of this study. Additionally, the decision to forego hyperparameter tuning and to adopt the original settings published by model developers suggests areas for future improvement.

This paper suggests that with expert-driven adjustments and advanced data analysis techniques, the accuracy of PHM systems in detecting and predicting failures could be significantly improved. Future research should aim at incorporating real operational data to validate the model’s effectiveness further and explore sophisticated preprocessing techniques to better address sensor data uncertainty. Optimizing model performance through tailored hyperparameter tuning could bridge the theoretical and practical applicability gap, enhancing the reliability and robustness of PHM models in operational environments.

## 6. Conclusions

This study emerges from the critical need to enhance the reliability and safety of cryogenic safety valves in the petroleum and LNG industries. Recognizing the limitations of traditional empirical methods and the scarcity of specific failure data for these valves, the research aims to develop a data-driven prognostics and health management (PHM) approach. By leveraging simulated failure data and comparing various time series analysis models, this study seeks to identify a predictive model that can accurately classify normal and abnormal operational states, thereby addressing a significant gap in the field.

In this study, the research methodology encompassed the acquisition of simulated failure data to replicate cryogenic valve failure scenarios, addressing the challenge of scarce failure data in PHM applications. This approach allowed for the creation of a rich dataset for analysis. Data preprocessing involved standard techniques to clean the data, including the removal of outliers and handling missing values, ensuring the quality and reliability of the data for model training. Various time series analysis models were then evaluated to determine their effectiveness in predicting valve failures. Experiments were designed to compare these models’ performance, focusing on their ability to distinguish between normal and abnormal operational states accurately.

This study meticulously constructed a testbed, simulating real-world conditions to gather comprehensive data under both normal and abnormal states, thereby overcoming the challenges of data scarcity in failure prediction. Through rigorous preprocessing and the application of sophisticated machine learning models, it was demonstrated that predictive models trained on normal operation data could effectively distinguish between normal and abnormal states, showcasing high precision and recall rates. Despite relying on simulated data and not performing extensive hyperparameter tuning, this research lays a strong foundation for future advancements in PHM systems, highlighting the potential for real-time anomaly detection and proactive maintenance strategies in industrial applications.

For future research, this study highlights the potential for enhancing predictive models’ accuracy and reliability by incorporating real operational data to validate and refine the predictive capabilities further. Investigating advanced data preprocessing techniques and custom hyperparameter tuning could address current limitations, improving the models’ ability to handle the complexity and variability of real-world data. Additionally, exploring the integration of expert insights for more precise anomaly detection thresholds and extending the model to cover a broader range of operational parameters and valve types could significantly contribute to the development of more robust, efficient, and adaptable PHM systems.

## Figures and Tables

**Table 1:** Comparison of short-term forecasting for pressure by model.

Model	MSE
XGBoost	2.116176 × 10^6^
LSTM	2.011905 × 10^6^
Inforemr	1.999571 × 10^6^
SCINet	1.965583 × 10^6^

**Table 2:** Model classification accuracy on mixed operational data using deviation threshold for anomaly detection.

	Precision	Recall	F1-Score	Support
Normal	0.9993	0.9988	0.9990	50,986
Abnormal	0.8492	0.8989	0.8734	376
macro average	0.9243	0.9489	0.9362	
micro average	0.9981	0.9981	0.9981	

## Data Availability

The data used in this manuscript are related to equipment malfunctions and are considered a significant asset of the company, making it challenging to disclose publicly. Therefore, we are unable to provide specific data supporting the results reported in this study due to privacy and ethical restrictions. We appreciate your understanding and cooperation.
